# The VapBC-4 Characterization Indicates It Is a Bona Fide Toxin-Antitoxin Module of *Leptospira interrogans*: Initial Evidence for a Role in Bacterial Adaptation

**DOI:** 10.3390/microorganisms13040879

**Published:** 2025-04-11

**Authors:** Bruna Oliveira Pigatto Azevedo, Deborah Kohn Damiano, Aline Florencio Teixeira, Ana Lucia Tabet Oller Nascimento, Luis Guilherme Virgilio Fernandes, Alexandre Paulo Yague Lopes

**Affiliations:** 1 Laboratório de Desenvolvimento de Vacinas, Instituto Butantan, São Paulo 05503-900, Brazil; brunapigatto@usp.br (B.O.P.A.); deborah.damiano@usp.br (D.K.D.); aline.rteixeira@fundacaobutantan.org.br (A.F.T.); ana.nascimento@butantan.gov.br (A.L.T.O.N.); 2 Programa de Pós-Graduação Interunidades em Biotecnologia, Instituto de Ciências Biomédicas, Universidade de São Paulo, São Paulo 05508-900, Brazil; 3 Infectious Bacterial Diseases Research Unit, Agricultural Research Service, US Department of Agriculture, Ames, IA 50010, USA; luis.fernandes@usda.gov

**Keywords:** toxin-antitoxin, VapBC, VapC, *Leptospira*

## Abstract

Toxin-antitoxin (TA) systems are one of the bacterial adaptation mechanisms to adverse conditions. *Leptospira interrogans* serovar Copenhageni contains nine putative TA systems. To date, only VapBC-3 and VapBC-1 have been experimentally characterized and considered functional modules. This study shows that the VapBC-4 module is a novel bona fide TA system constituted by VapB-4 antitoxin and VapC-4 toxin. Overexpression of the recombinant toxin in *Escherichia coli* resulted in growth inhibition, which was rescued by co-expression of the VapB-4 antitoxin. The toxin-antitoxin binding capability, essential to TA functionality, was demonstrated by dot blot assay in vitro, while the pull-down assay indicates that the toxin and antitoxin interact in vivo. In addition, we confirmed that VapC-4 is a PIN domain endoribonuclease capable of degrading viral MS2 substrate. The transcriptional studies suggest that *vapC-4* may be involved in the virulence and adaptability of *L. interrogans* serovar Copenhageni for adverse environmental conditions. Taken together, these results show that the VapBC-4 module is functional and can be considered a bona fide module.

## 1. Introduction

Toxin-antitoxin (TA) systems are considered modules for adapting bacteria to unfavorable environmental conditions. These genetic elements code for two components, a toxin that interferes with multiple cellular processes and its cognate antitoxin that inhibits toxicity [1,2,3]. TA systems are classified into eight types according to the molecular nature of the components, either protein or nucleic acid, and how the antitoxin interferes and neutralizes the effect of the toxin [1,4,5]. Type I features antisense RNA antitoxins that inhibit toxin mRNA translation [6,7]. Type II includes protein antitoxins that act as enzyme inhibitors [8]. Type III utilizes RNA antitoxins that form protein–-RNA complexes to block toxins [9]. Type IV antitoxins are proteins that block toxin binding by competing for the same substrate [10]. Type V features antitoxins with RNAse activity targeting toxin mRNA [11]. Type VI antitoxins are ATP-dependent proteases that degrade toxins [12]. Type VII antitoxins inhibit toxins via post-translational modification [13]. In Type VIII, both antitoxin and toxin are RNAs, with the antitoxin acting as an antisense RNA or transcriptional repressor [14]. Type II systems are the most abundant in bacteria and some archaea [15].

Type II TA modules are the most prevalent and are categorized into 14 different families based on the structure of the toxins and similarities in protein sequences. The VapBC (virulence-associated proteins B and C) [16] family is the largest within Type II TA modules, with more than 1900 VapBC modules identified across 960 genomes. This family accounts for 30 to 40% of all known toxin-antitoxin systems. VapCs, like the toxins from the RelBE, MazEF, and HicAB families, have been identified as endoribonucleases, also referred to as RNA interferases [15,17,18]. In the Type II TA system, antitoxins are proteins that interact with toxins as enzymes and inhibitors, and they are divided into families according to the structure of the toxin. In addition to being the most abundant and diverse, Type II TA modules are represented in most bacterial and archaeal genomes [15]. Besides binding to its cognate toxin, the antitoxin plays a role in regulating the expression of the Type II TA operon by binding to the operator, resulting in transcription repression. In most cases, complete repression is achieved in the presence of the TA complex, as toxin binding enhances the DNA binding capacity of the antitoxin [19].

Due to the unstable nature of the antitoxin and its susceptibility to degradation by host proteases related to stress conditions (e.g., Lon or Clp), the cleavage of antitoxin frees the stable toxin, altering the T:A ratio, which, in turn, releases the toxin into the cell to act on its cellular target. Therefore, antitoxin protein must be constantly replenished to avoid excess of toxin [19]. This explains the organization of most Type II TA operons: the antitoxin gene preceding the toxin gene, allowing the antitoxin to be transcribed and translated before toxin synthesis begins [20,21,22]. The function of the TA systems relates to several physiological processes that go beyond being involved in plasmid maintenance and programmed cell death [23]. It has been reported that they contribute to the bacterial regulatory machinery [24,25] and are associated with stress management, resulting in adaptation to environmental stress [26].

Type II TA systems have been shown to be involved in the stress response of bacterial pathogens, which can result in biofilm formation [27], dormancy [28], persistence [11], pathogenicity [29], resistance to phage infection [30,31], and regulation of gene expression [32,33]. Consequently, the stress response favors the bacteria survival in different ecosystems by detecting and adapting to changes in the environment and altering their gene expression patterns [1,4,34,35].

The investigation of bacterial genomes led to the discovery of many TA modules widespread in bacteria. In *L. interrogans* serovar Copenhageni, nine putative Type II TA modules were identified, four of which belong to the VapBC family [36]. *L. interrogans*, a pathogenic bacterium of the genus *Leptospira*, is an aerobic, tightly coiled spirochete [37] responsible for most cases of leptospirosis in humans in Brazil [38].

Leptospirosis is the most widespread zoonosis, responsible for over 1 million human cases and 60,000 deaths annually, with a higher prevalence in tropical and subtropical countries [39,40]. Human infection occurs mainly through the contact of exposed abraded skin or mucosa with the bacteria present in infected hosts’ biological fluids. Leptospirosis can be a self-limiting disease with flu-like symptoms; however, the disease can evolve into a severe condition with a high mortality rate known as Weil’s syndrome, corresponding to 5–15% of the reported cases, which causes kidney and liver dysfunction, and jaundice [41,42].

The TA VapBC system was first identified as a sequence of genes present in a plasmid prevalent in virulent isolates of the bacteria *Dichelobacter nodosus* [16], being the most abundant family Type II TA. Toxins from VapBC are described as endoribonucleases based on the presence of a PIN (PilT N-terminal) catalytic domain containing 4–5 conserved acidic residues, all of which are responsible for coordinating one or more divalent cations in the catalytic center [43,44,45].

In 2019, an in silico analysis study from our group reported the presence of four VapBC modules in the genome of *L. interrogans* serovar Copenhageni strain Fiocruz L1-130, which were named VapBC-1, -2, -3, and -4 based on their numerical order of appearance in the genome [36]. In the present study, the VapBC-4 module was experimentally characterized and investigated. We provided experimental evidence demonstrating the main defining characteristics of TA modules, such as bacterial growth arrest caused by overexpression of the toxin; in vivo and in vitro physical interaction between VapB-4 and VapC-4; ribonuclease activity of the toxin; and transcription studies showing the module is affected by nutritional stress. Altogether, the functional and structural results strongly indicate that VapBC-4 is a bona fide element.

## 2. Materials and Methods

### 2.1. Strains and Culture Conditions

The *E. coli* strain DH5α (ThermoFisher Scientific, Waltham, MA, USA) was used for cloning experiments and strain BL21 (DE3) (ThermoFisher Scientific, Waltham, MA, USA) was used for recombinant protein expression. The *E. coli* strains were cultured in Lysogeny Broth (LB) medium with 100 µg/mL kanamycin (Kan) supplemented with 1% glucose. *L. interrogans* serovar Copenhageni strain Fiocruz L1-130, and strain M20, provided by the bacterial zoonosis laboratory of the Faculty of Veterinary and Animal Science of the University of Sao Paulo (USP), were grown in EMJH medium (Difco, Franklin Lakes, NJ, USA) with 10% *v*/*v* Leptospira Enrichment EMJH (Difco, Franklin Lakes, NJ, USA) or in-house EMJH medium (Na_2_HPO_4_, KH_2_PO_4_, NaCl, 10% glycerol, 25% NH_4_Cl, supplemented with ZnSO_4_ 7H_2_O, CaCl_2_ 2H_2_O, MgCl_2_ 6H_2_O, thiamine, vitamin B12, MnSO_4_ 7H_2_O, FeSO_4_ 7H_2_O, Tween 80, pH 7.4) with 1% *v*/*v* rabbit serum, at 30 °C for approximately 7 days. *L. interrogans* serovar Copenhageni (M20) has been culture-attenuated by several passages since its isolation in 1938 from a patient in Denmark [46].

### 2.2. In Silico Studies

The sequences of the VapC-4 from *L. interrogans* and VapC from *L. kirschneri*, *L. santarosai*, *L. inadai* and *L wolbachii* were extracted from the data base “National Center for Biotechnology Information” (NCBI). Alignment of VapC-4 with other VapCs was performed by the SnapGene^®^ 6 software (Dotmatics, Boston, MA, USA). Protein domain study was analyzed by Pfam 37.2 (http://pfam.xfam.org/, accessed on 18 July 2024). For prediction of the tertiary (3D) structures, the server I-TASSER (https://zhanggroup.org/I-TASSER/, accessed on 15 October 2023) was used. The quality of modeling was evaluated in the PyMOL (The PyMOL Molecular Graphics System, Version 3.0 Schrödinger, LLC, accessed on 29 October 2024).

### 2.3. Genes and Plasmid Construction

The genes *vapB-4* (old locus tag: LIC_12712; new Gene ID: LIC_RS 13910) and *vapC-4* (old locus tag: LIC_12713; new Gene ID: LIC_RS 13915) were constructed adding a N-terminal 6xHis Tag, and full VapBC-4 module with a C-terminal 6xHis Tag from pET28a. The genes were amplified by PCR from genomic DNA of *L. interrogans* serovar Copenhageni strain Fiocruz L1-130, using the primers listed in Table 1. *Nco* I (forward primer) and *Xho* I (reverse primer) restriction sites and nucleotides coding a histidine tag (His6X) were incorporated. PCR products were cloned into pET28a (Novagen^®^, Merck KGaA, Darmstadt, Germany). The constructions were confirmed by DNA sequencing (ABI PE Applied Biosystems, ThermoFisher Scientific, Waltham, MA, USA) using primers T7 forward (5′ CCAAAGTGTTTTTAATCCATGTGATC 3′) and T7 reverse (5′ TATGCTAGTTATTGCTCAG 3′).

### 2.4. Protein Synthesis and Purification

The clones of *E. coli* BL21 cells transformed with pET*vapB-4*, pET*vapC-4* and pET*vapBC-4* were grown in LB/Kan medium at 37 °C. Gene expression was induced by 0.1 mM IPTG at OD_600_ of 0.6. The cells were cultured for 3 h at 37 °C for VapB-4 and complex VapBC-4 or overnight at 20 °C for VapC-4. The cells were harvested by centrifugation and resuspended in lysis buffer (50 mM Tris, 150 mM NaCl in pH 8.0) and cell pellets were lysed by a high-pressure homogenizer (Panda, GEA Niro Soavi, Parma, Italy). The pellets of the insoluble fraction were solubilized in 8 M urea, 500 mM NaCl, 50 mM Tris (pH 8.0) and then applied to Ni-Sepharose resin for purification of proteins by immobilized metal ion affinity chromatography (IMAC). The proteins’ refolding process was performed in the chromatography column by a decreasing urea gradient. After refolding, the proteins were eluted with elution buffer (50 mM Tris, 150 mM NaCl, 1 M imidazole in pH 8.0). The fractions recovered were dialyzed in the 3500 MWCO SnakeSkin^TM^ (ThermoFisher Scientific, Waltham, MA, USA) and quantified by bicinchoninic acid (BCA).

### 2.5. Production of VapB-4 Anti-Serum

Four female BALB/c mice, aged 1 to 2 months and weighing between 18 and 25 g, were used in this study. This number of animals is the minimum required to collect serum in adequate quantities. The mice were injected with four subcutaneous doses containing 5 µg of VapB-4, mixed with aluminum hydroxide (12.5%) as adjuvant, with 15 days of interval between each immunization. No animals were excluded from this study, as the experiment was solely designed to obtain anti-serum. Before immunization, the mice were bled from the retro-orbital plexus and serum was separated by centrifugation (3381× *g*, 15 min). Prior to bleeding, a drop of anesthetic eye drops (ANESTALCON^®^—proxymetacaine hydrochloride 0.5%) was administered via the eye. Negative control serum was collected from animals injected with PBS plus adjuvant. The production of IgG antibody was analyzed by ELISA (Enzyme-Linked Immunosorbent Assay) and validated by Western blotting assay with whole-cell lysates from *L. interrogans* serovar Copenhageni strain Fiocruz L1-130.

#### Ethical Statement

This study was performed according to the guidelines outlined by the Brazilian National Council for Control of Animal Experimentation (CONCEA), which follows international guidelines for animal welfare and the principles of the 3Rs. Experimental protocols comply with the ARRIVE guidelines and were approved by the Ethic Committee on Animal Use of the Butantan Institute, São Paulo, Brazil (protocol number 3200120118). The mice were housed in a BSL2 animal facility in micro isolators with individual ventilation and temperature and light cycle control. The animals received food and water ad libitum, and manipulation was performed by trained personnel.

### 2.6. E. coli Growth Kinetics

*E. coli* BL21 (DE3) was transformed with the plasmid constructions pET*vapB-4*, pET*vapC-4* and pET*vapBC-4*, cultivated in 50 mL of LB/Kan with 1% glucose at 37 °C until OD_600_ of 0.1 and induced by addition of 0.1 mM IPTG to the medium. After induction, optical density (600 nm) was measured every hour for five hours. *E. coli* cells containing empty pET28a were used as a control.

### 2.7. Pull-Down Assay

The soluble fraction of *E. coli* BL21 (DE3) containing pET*vapBC-4* after IPTG induction was submitted to IMAC, as described above (Section 2.4). The eluted fractions were analyzed by SDS-PAGE to test the interaction between the co-expressed recombinant proteins VapB-4 and VapC-4.

### 2.8. Affinity Dot Blot Assay

Recombinant toxin VapC-4 (2 µg) was adsorbed in triplicate on a nitrocellulose membrane (BioRad, Hercules, CA, USA). The protein spots on the membrane were dried at room temperature and the membrane was blocked overnight with 10% non-fat dry milk in PBS-Tween 20 (PBS-T), washed three times for five min with PBS-T and incubated with 3 µg/mL of recombinant VapB-4 protein in PBS for 2 h. The membranes were washed again and incubated with polyclonal antibodies against VapB-4, diluted 1:1000 in 10% non-fat dry milk in PBS-T, for 1 h. After three washes, the membrane was incubated with horseradish peroxidase (HRP)-conjugated secondary anti-mouse IgG antibodies (Invitrogen-ThermoFisher Scientific, Waltham, MA, USA) diluted 1:1000 in 10% non-fat dry milk in PBS-T, for 1 h. The membrane was further washed and developed by chemiluminescence using SuperSignal^®^ West Dura Extended Duration (Invitrogen-ThermoFisher Scientific, Waltham, MA, USA). The image was visualized in Amersham Imager 600 (GE Healthcare, Chicago, IL, USA).

### 2.9. Ribonuclease Activity Assay

Viral MS2 RNA (Roche, Basel, Switzerland) was used for ribonuclease activity assay. Different toxin concentrations (0.5 µg; 1 µg; 2 µg; 3 µg) were incubated with RNA MS2 substrate in 300 mM Tris-HCl buffer pH 7.0 in the presence of 0.1 mM MgCl^2+^ by 2 h, at 37 °C. VapC-4 (1 µg) inhibition by VapB-4 (1 µg; 2 µg; 3 µg) was also tested by preincubating VapB-4 and VapC-4 before adding MS2. The results were analyzed in 10% Ureia-TBE gel (Invitrogen-ThermoFisher Scientific, Waltham, MA, USA), stained with SYBR^®^ Safe DNA (Invitrogen-ThermoFisher Scientific, Waltham, MA, USA) in a proportion 1:10,000 on TBE buffer and revealed on a transilluminator (312 nm).

### 2.10. Transcription Studies by RT-qPCR

To measure *vapB-4*, *vapC-4*, and *vapBC-4* transcription, cultures from *L. interrogans* serovar Copenhageni strain Fiocruz L1-130 (virulent) and strain M20 (culture-attenuated) were harvested by centrifugation after being grown until 10^8^ cells. Total RNA was extracted and converted into cDNA using SuperScript^®^ III First-Strand Synthesis System (Invitrogen-ThermoFisher Scientific, Waltham, MA, USA). The gene expression was evaluated by qPCR using C1000 Touch™ Thermal Cycler (Bio-Rad, Hercules, CA, USA). The reaction used the cDNA, primers (Table 1) and Power SYBR^®^ Green PCR Master Mix (Applied Biosystem-ThermoFisher Scientific, Waltham, MA, USA). The 16s gene was used as the control.

To study the influence of nutritional stress on the activation of the TA system, *L. interrogans* serovar Copenhageni cultures were subjected to nutrient deprivation by replacing the in-house EMJH medium with PBS (phosphate-buffered saline pH 7.4). Leptospiral cultures were grown to reach 10^8^ cells and subsequently pelleted via centrifugation at 11.5× *g* for 15 min. The supernatant was discarded and one culture was resuspended in PBS (nutrient-depleted medium for leptospiral growth) and the control was resuspended in traditional medium (in-house EMJH). The cultures were kept under stress for 24 h, at 30 °C. After this period, the cultures were centrifuged and processed for RNA extraction, cDNA preparation and RT-qPCR analysis to evaluate the relative transcription of the *vapC-*4 gene and *lipL32* as another target gene. The relative gene expression levels were assessed according to the 2^−ΔΔCt^ method using 16s as reference gene.

## 3. Results

### 3.1. The VapBC-4 Module from L. interrogans

**Figure 1:**
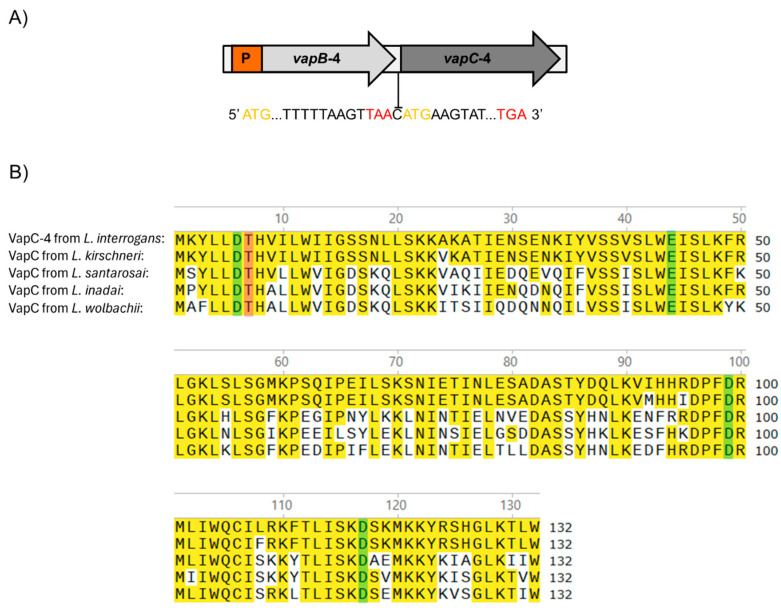
(**A**) Schematic representation of the VapBC-4 module of *L. interrogans* serovar Copenhageni strain Fiocruz L1-130. Yellow and red letters represent start and stop codons, respectively. P is the promoter. The two genes are separated by one cytosine in a typical structure in which the phenomenon of “translation coupling” occurs. (**B**) Alignment of the sequences of VapC-4 from *L. interrogans* with VapC of *L. kirschneri*, *L santarosai*, *L. inadai* and *L. wolbachii*. Highlighted in yellow are the identical amino acids, and in green and pink are the four acidic residues and the invariant threonine essential for the catalytic activity of the PIN domain, respectively. Alignment was performed with the SnapGene 6 software using Clustal Omega tool.

The *vapBC*-4 *locus* is localized in chromosome I of pathogenic *L. interrogans* serovar Copenhageni strain Fiocruz L1-130 [47] and organized as a bicistronic operon composed of an upstream 234 bp *vapB-4* antitoxin gene (LIC_12712) and a downstream 399 bp *vapC-4* toxin gene (LIC_12713), resulting in VapB-4 and VapC-4 proteins with 77 and 132 amino acids, respectively. The two genes are separated by an intergenic spacer constituted by a unique cytosine, indicating translational coupling (Figure 1A).

The protein domain analysis using Pfam webserver [48] indicated that the hypothetical protein VapC-4 functions as a ribonuclease. The alignment of the protein primary sequences showed the presence of a conserved four-amino acid sequence and an invariant threonine, characteristic of PIN domain present in the VapC toxins [43]. BLAST 1.4.0 analysis showed that, among the four VapBC modules of *L. interrogans* serovar Copenhageni, VapC-4 is the second most extensively distributed among *Leptospira* species, being highly conserved only in *L. kirschneri* (97%), followed by *L. santarosai* (64%), *L. inadai* (65%), and *L. wolbachii* (64%) [36]. The sequence alignment among these species showed the conservation of 4 acidic residues responsible for coordinating Mg^2+^ or Mn^2+^ ions at the catalytic site (Figure 1B).

**Figure 2:**
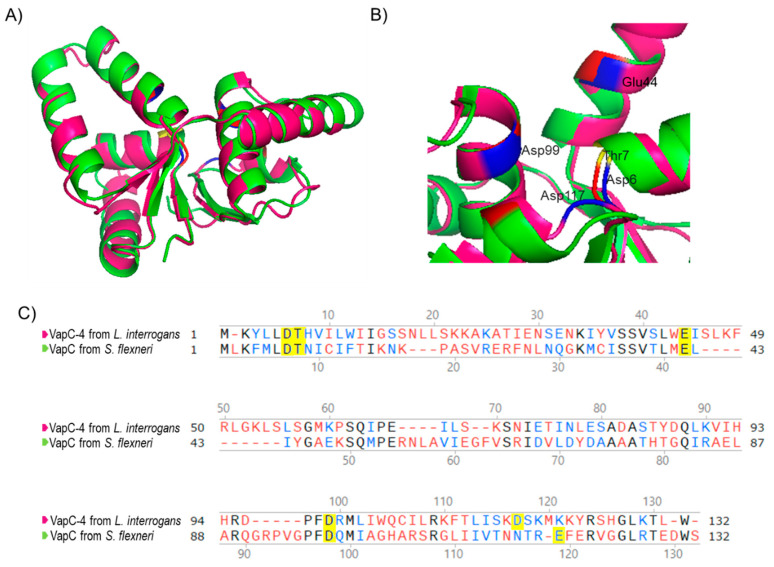
Three-dimensional model structure of VapC-4 constructed with I-TASSER and PyMOL tools. (**A**) Superposition of the tertiary structures of VapC-4 from *L. interrogans* (pink) and VapC from *S. flexneri* (green) (PdB: 3TND-C), blue and red indicate the acidic amino acids overlaping and yellow the threonine. (**B**) PIN domain conserved residues: Asp6, Thr7, Glu44, Asp99 and Asp117. (**C**) Alignment of the sequences of VapCs from *S. flexneri* and *L. interrogans*. Amino acids colored in black are identical, in blue are similar and in red are non-similar. The yellow-highlighted amino acids represent the Pin domain conserved residues.

Considering that the 3D structure of an enzyme correlates with its biochemical activity, the primary sequence of VapC-4 from *L. interrogans* was submitted to 3D modeling and alignment (Figure 2) using as a template the experimentally solved X-ray structure of VapC from *Shigella flexneri* [49]. The in silico structural model (Figure 2A) shows that β-sheets are present in the center of the structure, surrounded by α-helixes, and the conserved residues responsible for coordinating metal ions in the catalytic site are shown in detail: Asp6, Thr7, Glu44, Asp99, and Asp117 (Figure 2B). According to the structural conservation of the PIN domain model, despite the diverse primary sequences (Figure 2C), VapC-4 denotes the necessary features to be an active ribonuclease.

### 3.2. Production of Recombinant VapB-4 and VapC-4 Proteins Independently and in Tandem

**Figure 3:**
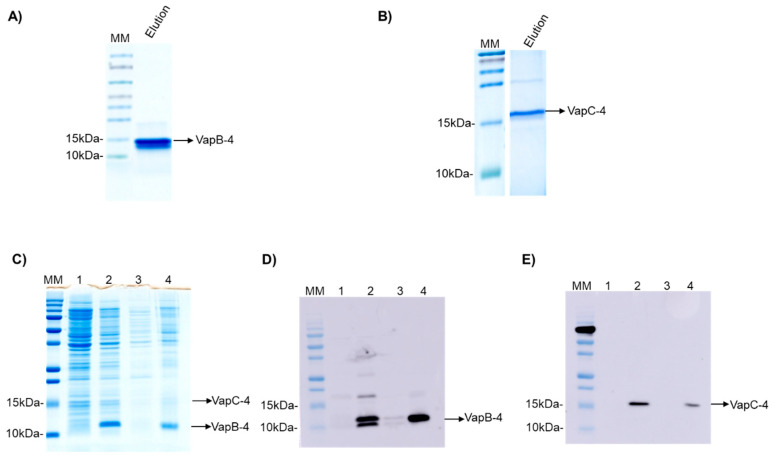
Analysis of purified recombinant proteins by SDS-PAGE (20%) and Western blotting. (**A**) Eluted fraction of recombinant VapB-4. (**B**) The eluted fraction of recombinant VapC-4. (**C**) Analysis of expression of VapBC-4 module by SDS-PAGE. (**D**) Western blotting using anti-VapB-4 antibody. (**E**) Western blotting using anti-6X-His antibody. Lanes: (MM) molecular marker; (1) non-induced extract; (2) induced extract; (3) soluble fraction; (4) insoluble fraction.

The operon *vapBC-4* genes were cloned under the control of a single promoter in the pET28a vector. The proteins were synthesized in *E. coli* BL21 (DE3) and displayed an apparent molecular weight of 9.5 and 16.2 kDa for VapB-4 and VapC-4, respectively (Figure 3A,B). The synthesis of the toxin and antitoxin in tandem as a full operon is important to the characterization of the operon functionality and indicates that the bicistronic mRNA was processed the same way it is processed in *Leptospira.* The production of the proteins in complex was evaluated by SDS-PAGE (Figure 3C) and Western blotting. The antibodies Anti-VapB-4 and anti-His were used to confirm the presence of the VapB-4 protein and the VapC-4 protein, respectively (Figure 3D,E). When proteins are produced in tandem VapC-4 is generated in a minor amount than Vap-4, possibly because, as it commonly happens in TA operons, the antitoxin gene is followed by the toxin gene in a genetic arrangement where there is a space or overlap between genes, leading to translational coupling. In this kind of phenomenon, it is possible to occur the decoupling of the translational machinery, causing differences in the proportion of the synthesis of the two proteins [50].

### 3.3. VapC-4 Toxin Inhibits E. coli Growth Rate

**Figure 4:**
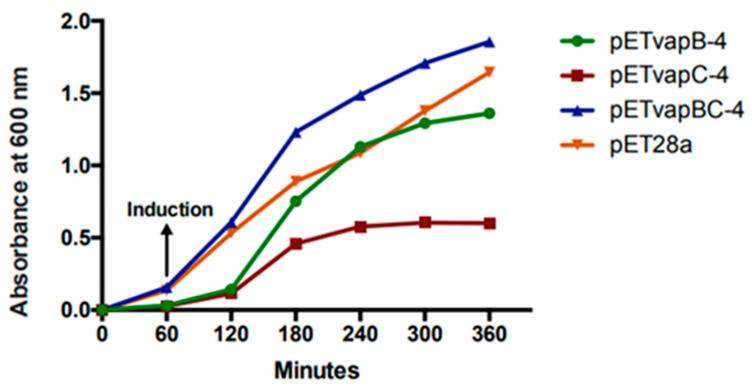
Evaluation of VapC-4 and VapB-4 influence on the growth rate of *E. coli* transformed with the constructions pETvapB-4, pETvapC-4, pETvapBC-4, and empty pET28a. Optical density was measured for 5 h after IPTG induction.

To evaluate how a heterologous toxin from a TA module affects *E. coli* kinetics, the growth of *E. coli* containing pET-*vapB-4*, pET-*vapC-4*, pET-*vapBC-4*, and empty vector was monitored on a timely basis. The effect of VapC-4 overexpression showed significant inhibition of the growth of *E. coli* BL21 (DE3) after IPTG induction (Figure 4). There was no significant difference in the tendency of growing curves of the constructs containing the *vapB-4* gene and the empty vector, which also happened with *E. coli* co-expressing the antitoxin and toxin, evidencing that the toxicity displayed by the toxin VapC-4 was neutralized by the antitoxin VapB-4. Non-induced controls grew exponentially as expected. Looking in detail, there is a growth of the clone pET*-vapC-4* in the first 2 h, although slow compared to growth of the clone pET-*vapBC-4*. The pETvapC-4 clone stops growing at an optical density (O.D.) of 0.5, while the pET-*vapBC-4* clone, which expresses both VapB-4 and VapC-4, grows to nearly 2.0 O.D.

### 3.4. The Antitoxin VapB-4 Binds to the Toxin VapC-4 In Vivo and In Vitro

**Figure 5:**
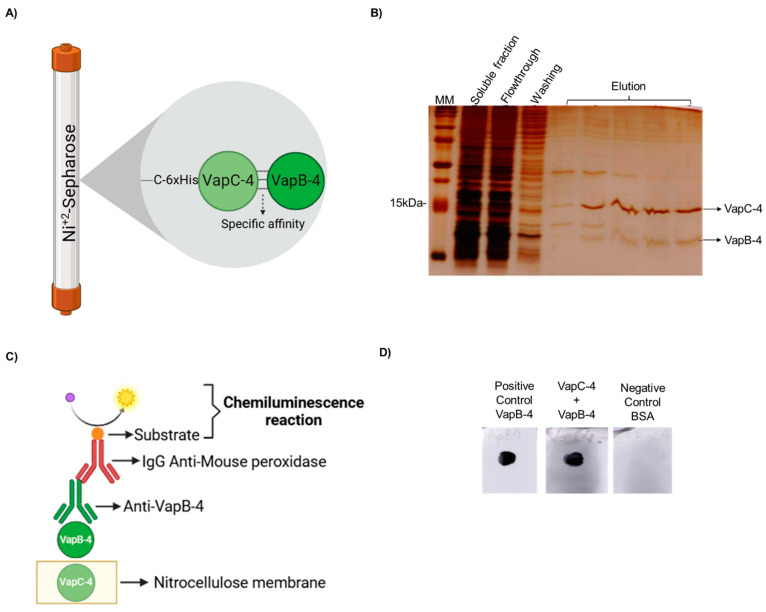
VapB-4 and VapC-4 bound in vivo and in vitro. (**A**) Diagram showing specific affinity of VapB to 6xHis-VapC attached to Ni^+2^ by the pull-down assay. (**B**) Analysis by SDS-PAGE (20%) of the pull-down assay of the soluble fraction of *vapBC-4* culture evidencing the in vivo interaction. Lanes: MM; soluble fraction; flowthrough; washing; elution fractions of VapBC-4 module. (**C**) Schematic diagram of dot blot assay for testing the specific binding of recombinant VapB and VapC. (**D**) Lanes: positive control: recombinant VapB-4 adsorbed to the membrane; recombinant VapC-4 attached to the membrane incubated with VapB-4; negative control: BSA protein adsorbed to the membrane. This result shows the in vitro interaction. (**A**,**C**) Created with BioRender.com. (https://app.biorender.com/, accessed on 30 January 2025).

A typical feature of Type II TA systems is the direct interaction of the toxin with the antitoxin and the formation of a protein complex in vivo. To demonstrate it, a pull-down assay was performed. The proteins were produced in complex in *E. coli* BL21 (DE3) containing pET-*vapBC-4*, and the soluble fraction of this culture was purified by IMAC. It is possible that both proteins of the complex are co-purified in the elution fractions (see the scheme in Figure 5A), which shows that the nontagged VapB-4 was able to interact with VapC-4. The toxin-antitoxin interaction is suggestive that it is the way VapB-4 neutralized the toxic effect of VapC-4 (Figure 5B).

The in vitro interaction between VapC-4 and VapB-4 was validated by a dot blot assay (Figure 5C). In this method, the membrane was labeled with VapC-4 and incubated with a VapB-4 solution. The membrane was incubated with anti-VapB-4 antiserum and then with a secondary antibody (anti-mouse IgG-peroxidase) and revealed by chemiluminescence. The results showed an interaction between VapC-4 and VapB-4 (Figure 5D).

### 3.5. Determination of VapC-4 Ribonuclease Activity

**Figure 6:**
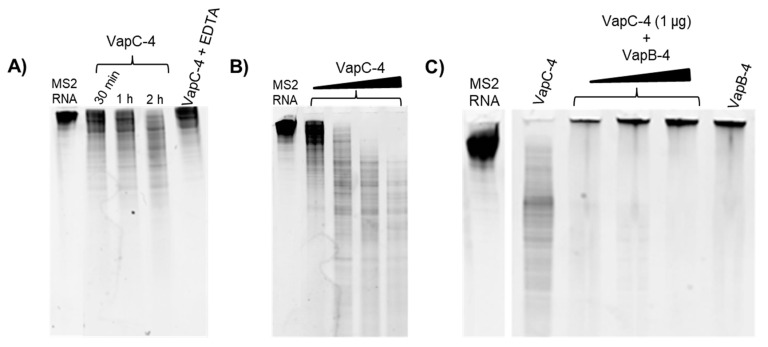
Analysis of the ribonuclease activity of the toxin VapC-4 in TBE urea PAGE (10%). (**A**) Time dependency curve of VapC-4 (1.0 µg) towards MS2 RNA (1.0 µg) in the presence of 0.1 mM Mg^2+^ by 0.5, 1 and 2 h incubation and activity abrogation by EDTA (10 mM). (**B**) VapC-4 dose dependence curve (0.5 µg; 1.0 µg; 2.0 µg; 3.0 µg) on MS2 RNA substrate. (**C**) Inhibition of VapC-4 (1.0 µg) by VapB-4 (1.0, 2.0 and 3.0 µg) towards MS2 RNA.

To investigate whether VapC-4 exhibits ribonuclease catalytic activity as predicted for PIN domain proteins, we tested VapC-4 activity using MS2 RNA as substrate. The optimal concentration of the cofactor Mg^2+^ was defined as 0.1 mM. In order to confirm the divalent metal dependence of the enzyme, we tested EDTA, whose chelating effect inhibited the degradation of MS2 (Figure 6A). As shown in Figure 6A,B, VapC-4 efficiently degraded MS2 RNA in a time-dependent and dose-dependent manner. Although the inhibition of the VapC-4 by VapB-4 could have occurred, this effect was not observed (Figure 6C). One possibility is that the incubation of antitoxin and substrate produced an interaction that did not allow the sample to migrate through the gel, as seen in all the samples where VapB-4 is incubated with MS2.

### 3.6. Analysis of vapC-4 Transcription

**Figure 7:**
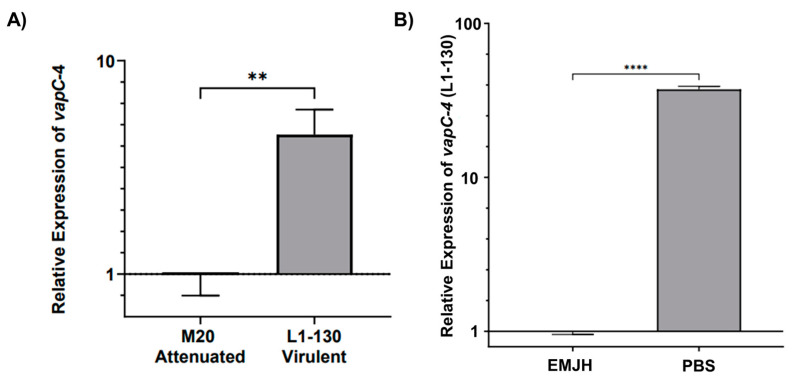
Relative expression analysis of *vapC-4* measured by qPCR. (**A**) Attenuation effect on transcription (*L. interrogans* serovar Copenhageni Fiocruz L1-130 to the attenuated strain M20). (**B**) Evaluation of nutritional stress on *vapC-4* relative expression under normal conditions (EMJH) and under nutritional stress conditions (PBS). The relative gene expression levels were assessed according to the 2^−ΔΔCt^ method using 16s as reference gene in both tests. The data were transformed using logarithm base-10, and statistical analysis was performed using an unpaired t-test, followed by Welch’s post-test. A *p*-value of <0.01 (**) and <0.0001 (****) was considered statistically significant, using the significance interval of 95%, mean ± SEM.

The transcript levels of *vapC*-4 were analyzed by qPCR in virulent *L. interrogans* serovar Copenhageni strain Fiocruz L1-130 and culture-attenuated strain M20 under normal and under nutritional stress conditions. When comparing whether the attenuation of *L. interrogans* affects *vapC*-4 transcription, we observed that the relative expression of the gene is 4.5 times higher (calculated using the 2^−ΔΔCt^ method*)* in the virulent strain than in the attenuated one, and it is statically significant (Figure 7A). It is known that toxins are activated upon nutritional starvation [51]; so, we tested this condition over the virulent *L. interrogans* strain Fiocruz L1-130, changing the culture from EMJH media to PBS. Alongside *vapC-4*, we also assessed *lipL32* as another target gene, a membrane lipoprotein and the most abundant protein in *L. interrogans*. Transcriptional analysis revealed *vapC-4* transcription was significantly up-regulated 37.3-fold versus control (Figure 7B), whereas *lipL32* showed a smaller 2.7-fold increase. Nutrient starvation did not reduce the viable cell count of *Leptospira*, but decreased cell motility after 24 h. Based on the available data, this decline in motility cannot be solely attributed to the activation of the VapBC module; notwithstanding, one of the known attributions to TA systems is to induce dormancy. These results might suggest some involvement of this TA module in bacterial virulence, as well as in the adaptability of *L. interrogans* serovar Copenhageni strain Fiocruz L1-130 in adverse environmental conditions.

## 4. Discussion

TA systems are encoded by ubiquitous genetic modules in prokaryotes and archaea. Although there is still controversy over the ultimate role of these TA modules, it is consensual that antitoxins are degraded in response to environmental stresses, and the toxins are released to act [52]. In free-living prokaryotes, TA systems are often present in large numbers, with bacteria typically containing more than one TA module belonging, many times, to the same TA family [3], as is the case of *L. interrogans* [36,53]. Notwithstanding, whether these TA modules play unique or redundant biological roles remains unclear. Therefore, it is important to identify and characterize if these modules are genuine elements.

Genomic analysis of the human pathogen *L. interrogans* serovar Copenhageni revealed the presence of nine TA systems, with four being different putative *vapBC loci* on chromosome I [36], which were initially predicted by the TADB tool (https://bioinfo-mml.sjtu.edu.cn/TADB2/index.php, assessed on 28 August 2018). VapBC-3 was previously characterized by our group [54], and recently, we experimentally characterized the VapBC-1 module [22], both of which are considered bona fide functional elements.

In the present study, we confirmed that the *vapBC-4* operon is organized with the *vapB-4* gene upstream and the *vapC-4* gene downstream with one base distance between them, which indicates translational coupling. This pattern is found in many operons that encode Type II TA modules and usually presents nucleotides that separate or overlap the operon cistrons [50]. According to our previous studies using the NCBI BLAST 1.4.0, the distribution and conservation of amino acids in the four TA modules of *L. interrogans* revealed, through sequence alignment of the four VapCs, a high degree of diversity and low identity among their primary amino acid sequences. However, the set of three or four acidic residues responsible for coordinating Mg^2^⁺ or Mn^2^⁺ ions at the catalytic site was conserved, which characterizes the PIN domain [36]. Moreover, the VapC-4 protein has a homolog that is nearly identical in *L. kirschneri* (97% identity) and similar to *L. santarosai*, *L. inadai*, and *L. wolbachii* presenting 64%, 65%, and 64% identity, respectively, among pathogenic, intermediate, and saprophytic species [36]. Thus, corroborating the idea that TA *loci* are acquired by horizontal gene transfer [55,56].

TA toxins are notably known for their toxicity not only to homologous bacteria but also to other heterologous prokaryotic cells, a characteristic commonly utilized in the study of TA modules [15,57,58]. Here, we show that heterologous expression of *vapC-4* in *E. coli* induced a clear inhibition of bacterial growth, evidencing the toxicity of VapC-4 from *L. interrogans*, which was restored by the expression of the cognate antitoxin. In our study, the VapC-4 clone did not cease growth immediately following induction. In fact, Song et al. (2023) discuss how the rate of growth inhibition varies among different toxins. Their article, which examines five TA modules from *Pseudomonas aeruginosa*, suggests that this variation may be due “to direct interference at the post-transcriptional level”, leading to an insufficient amount of active toxin initially available to stop growth immediately [30].

A crucial criterion for the characterization of Type II TA systems is the formation of a stable complex between the toxin and the antitoxin, which, in normal conditions, prevents VapC from acting [59]. The expression of VapBC-4 full operon in *E. coli* showed that the toxin and antitoxin were synthesized in tandem in the same way as they are probably expressed in *Leptospira*. Using a pull-down assay, VapB-4 was successfully co-purified with 6xHis-VapC-4 immobilized on Ni-Sepharose, indicating the ability of in vivo interaction between the two proteins. This method has been widely used to verify in vivo interaction between toxins and cognate antitoxins [19,22,60,61,62,63,64]. Furthermore, the binding between the recombinant toxin and the antitoxin was also demonstrated in vitro by the dot blot assay.

Numerous toxins have the function to cleave, degrade, or enzymatically alter their cellular targets, thus disturbing bacterial physiology, even when present in low protein concentrations [65]. The toxins of the VapBC family contain the PIN (N-terminal PilT) domain, which confers the ribonuclease activity to these toxins [43,66]. The catalytic activity of the recombinant VapC-4 protein was tested using the MS2 RNA substrate, and its ability to degrade RNA was observed, demonstrating its role as a ribonuclease, which is dependent on metal ions since its activity was inhibited in the presence of EDTA. Therefore, our results show that VapBC-4 is a bona fide element, like VapBC-1 [22] and VapBC-3 [54] from *L. interrogans* serovar Copenhageni.

Throughout their life cycles, bacteria must sense and respond to environmental stress, which may require drastic cellular reprogramming at the transcriptional and translational levels [67]. The study of TA systems has gained attention in recent years due to their essential role in regulating bacterial growth and promoting survival under stressful conditions [59]. Like our findings for the transcription of the *vapC-*4, nutrient starvation stress in cultured *E. coli* MC1000 also did not reduce viable cell counts, but the *relBE* promoter TA module was strongly activated during amino acids’ starvation, increasing *relBE* transcription without causing cell death [68]. Interestingly, there was no significant effect of nutrient starvation on the number of CFUs detected in any of the examined stages of *E. coli.* strain BA1250. Remarkably, the upregulation of transcriptional levels has been described in several Type II TA modules, such as the *yoeB-yefM* genes of *E. coli*. BA1250 [69], *vapBC*6, *vapBC*8, and *vapBC*22 of *Sulfolobus solfataricus* [52], among others. In *L. interrogans*, nutrient starvation showed a very significant increase in *vapC-4* transcripts, suggesting that the presence of this module positively influences the bacterium’s ability to adapt to challenging environments.

This study characterized the VapBC-4 module of *L. interrogans* serovar Copenhageni, demonstrating that it exhibits the typical features of a Type II TA system. Therefore, it can be considered a bona fide element that may participate in the bacterium’s adaptation to the host’s environment. The comparison of transcription of *vapC*-4 between the attenuated *L. interrogans* (M20) with the virulent one (L1-130) showed a significant reduction in the attenuated one, which indicates it can play a role on survival of the virulent *L. interrogans*. Moreover, the qPCR preliminary analysis on the nutrient deprivation suggest it can play an adaptive role in the bacterium’s ability to cope with nutrient starvation stress. These two observations enlighten the possible role on the adaptative character of this TA module, which we intend to further study. Thus, this work contributed to the goal of expanding the repertoire of functional TA modules in *L. interrogans* serovar Copenhageni.

## Figures and Tables

**Table 1:** Primer sequences used in this study.

Primer	Sequence (5′ → 3′)	Restriction Site	Construction
F-vapB4-Nhis ^1^	gagccatgggc**CACCACCACCACCACCAC**ATGAAATCGTATCCGGTTGG	*Nco* I	pET-*vapB-4*
R-vapB4-Nhis ^1^	gagcctcgagTGTTAACTTAAAAACTCTTCTTCGG	*Xho* I	
F-vapC4-Nhis ^1^	gagccatgggc**CACCACCACCACCACCAC**ATGAAGTATTTGCTTGATAC	*Nco* I	pET-*vapC*-*4*
R-vapC4-Nhis ^1^	gagcctcgagttaTCACCAAAGTGTTTTTAATCCATG	*Xho* I	
F-vapBC4-Chis ^1^	gagccatgggcATGAAATCGTATCCGGTTGG	*Nco* I	pET-*vapBC*-*4*
R-vapBC4-Chis ^1^	gagcctcgagCCAAAGTGTTTTTAATCCATGTGATC	*Xho* I	
F-qPCR-vapB-4 ^2^	CCGGTTGGCGAGCTTAAATC	-	-
R-qPCR-vapB-4 ^2^	TTACCTTTCCATCCAATAATCCT	-	-
F-qPCR-vapC-4 ^2^	ACGTTAGCTCAGTTTCTCTTTGG	-	-
R-qPCR-vapC-4 ^2^	ATCGTATGTGCTTGCATCGG	-	-
F-qPCR-vapBC-4 ^2^	GCCGATTGCCATGATTATTCCT	-	-
R-qPCR-vapBC-4 ^2^	AGATGAACCGATTATCCAAAGAA	-	-

^1^ Restriction enzymes recognition sites are underlined, histidine tag sequence is presented in bold upper-case letters followed by coding region sequences. ^2^ Primers using in qPCR.

## Data Availability

The original contributions presented in the study are included in the article/original images material, further inquiries can be directed to the corresponding author.

## References

[1] Chapartegui-González I., Khakhum N., Stockton J.L., Torres A.G. (2022). Evaluating the Contribution of the Predicted Toxin-Antitoxin System HigBA to Persistence, Biofilm Formation, and Virulence in Burkholderia pseudomallei. Infect. Immun..

[2] Jurėnas D., Fraikin N., Goormaghtigh F., Van Melderen L. (2022). Biology and evolution of bacterial toxin-antitoxin systems. Nat. Rev. Microbiol..

[3] Pandey D.P., Gerdes K. (2005). Toxin-antitoxin loci are highly abundant in free-living but lost from host-associated prokaryotes. Nucleic Acids Res..

[4] Page R., Peti W. (2016). Toxin-antitoxin systems in bacterial growth arrest and persistence. Nat. Chem. Biol..

[5] Bendtsen K.L., Brodersen D.E. (2017). Higher-Order Structure in Bacterial VapBC Toxin-Antitoxin Complexes. Subcell. Biochem..

[6] Fozo E.M., Makarova K.S., Shabalina S.A., Yutin N., Koonin E.V., Storz G. (2010). Abundance of type I toxin-antitoxin systems in bacteria: Searches for new candidates and discovery of novel families. Nucleic Acids Res..

[7] Fozo E.M., Hemm M.R., Storz G. (2008). Small toxic proteins and the antisense RNAs that repress them. Microbiol. Mol. Biol. Rev..

[8] Ramisetty B.C.M., Santhosh R.S. (2017). Endoribonuclease type II toxin-antitoxin systems: Functional or selfish?. Microbiology.

[9] Fineran P.C., Blower T.R., Foulds I.J., Humphreys D.P., Lilley K.S., Salmond G.P. (2009). The phage abortive infection system, ToxIN, functions as a protein-RNA toxin-antitoxin pair. Proc. Natl. Acad. Sci. USA.

[10] Masuda H., Tan Q., Awano N., Yamaguchi Y., Inouye M. (2012). A novel membrane-bound toxin for cell division, CptA (YgfX), inhibits polymerization of cytoskeleton proteins, FtsZ and MreB, in *Escherichia coli*. FEMS Microbiol. Lett..

[11] Wang X., Lord D.M., Cheng H.Y., Osbourne D.O., Hong S.H., Sanchez-Torres V., Quiroga C., Zheng K., Herrmann T., Peti W. (2012). A new type V toxin-antitoxin system where mRNA for toxin GhoT is cleaved by antitoxin GhoS. Nat. Chem. Biol..

[12] Aakre C., Phung T., Huang D., Laub M. (2013). A Bacterial Toxin Inhibits DNA Replication Elongation Through a Direct Interaction with the β Sliding Clamp. Mol. Cell..

[13] Marimon O., Teixeira J.M., Cordeiro T.N., Soo V.W., Wood T.L., Mayzel M., Amata I., García J., Morera A., Gay M. (2016). An oxygen-sensitive toxin-antitoxin system. Nat. Commun..

[14] Choi J.S., Kim W., Suk S., Park H., Bak G., Yoon J., Lee Y. (2018). The small RNA, SdsR, acts as a novel type of toxin in Escherichia coli. RNA Biol..

[15] Gerdes K., Christensen S.K., Løbner-Olesen A. (2005). Prokaryotic toxin-antitoxin stress response loci. Nat. Rev. Microbiol..

[16] Katz M.E., Strugnell R.A., Rood J.I. (1992). Molecular characterization of a genomic region associated with virulence in Dichelobacter nodosus. Infect. Immun..

[17] Sevin E.W., Barloy-Hubler F. (2007). RASTA-Bacteria: A web-based tool for identifying toxin-antitoxin loci in prokaryotes. Genome Biol..

[18] Xie Y., Wei Y., Shen Y., Li X., Zhou H., Tai C., Deng Z., Ou H.Y. (2018). TADB 2.0: An updated database of bacterial type II toxin-antitoxin loci. Nucleic Acids Res..

[19] Chan W.T., Espinosa M., Yeo C.C. (2016). Keeping the Wolves at Bay: Antitoxins of Prokaryotic Type II Toxin-Antitoxin Systems. Front. Mol. Biosci..

[20] Gerdes K., Maisonneuve E. (2012). Bacterial persistence and toxin-antitoxin loci. Annu. Rev. Microbiol..

[21] Muthuramalingam M., White J.C., Bourne C.R. (2016). Toxin-Antitoxin Modules Are Pliable Switches Activated by Multiple Protease Pathways. Toxins.

[22] Damiano D.K., Azevedo B.O.P., Fernandes G.S.C., Teixeira A.F., Gonçalves V.M., Nascimento A.L.T.O., Lopes A.P.Y. (2024). The Toxin of VapBC-1 Toxin-Antitoxin Module from. Microorganisms.

[23] Wen Y., Behiels E., Devreese B. (2014). Toxin-Antitoxin systems: Their role in persistence, biofilm formation, and pathogenicity. Pathog. Dis..

[24] Ogura T., Hiraga S. (1983). Mini-F plasmid genes that couple host cell division to plasmid proliferation. Proc. Natl. Acad. Sci. USA.

[25] Ren D., Bedzyk L.A., Thomas S.M., Ye R.W., Wood T.K. (2004). Gene expression in *Escherichia coli* biofilms. Appl. Microbiol. Biotechnol..

[26] Barth V.C., Chauhan U., Zeng J., Su X., Zheng H., Husson R.N., Woychik N.A. (2021). VapC4 toxin engages small ORFs to initiate an integrated oxidative and copper stress response. Proc. Natl. Acad. Sci. USA.

[27] Kim Y., Wang X., Ma Q., Zhang X.S., Wood T.K. (2009). Toxin-antitoxin systems in Escherichia coli influence biofilm formation through YjgK (TabA) and fimbriae. J. Bacteriol..

[28] Moreno-Del Álamo M., Marchisone C., Alonso J.C. (2020). Antitoxin ε Reverses Toxin ζ-Facilitated Ampicillin Dormants. Toxins.

[29] Georgiades K., Raoult D. (2011). Genomes of the most dangerous epidemic bacteria have a virulence repertoire characterized by fewer genes but more toxin-antitoxin modules. PLoS ONE.

[30] Song Y., Tang H., Bao R. (2023). Comparative analysis of five type II TA systems identified in. Front. Cell Infect. Microbiol..

[31] Song S., Wood T.K. (2020). A Primary Physiological Role of Toxin/Antitoxin Systems is Phage Inhibition. Front. Microbiol..

[32] Hayes F., Kędzierska B. (2014). Regulating toxin-antitoxin expression: Controlled detonation of intracellular molecular timebombs. Toxins.

[33] Bloom-Ackermann Z., Steinberg N., Rosenberg G., Oppenheimer-Shaanan Y., Pollack D., Ely S., Storzi N., Levy A., Kolodkin-Gal I. (2016). Toxin-Antitoxin systems eliminate defective cells and preserve symmetry in *Bacillus subtilis* biofilms. Environ. Microbiol..

[34] Zadeh R.G., Kalani B.S., Ari M.M., Talebi M., Razavi S., Jazi F.M. (2022). Isolation of persister cells within the biofilm and relative gene expression analysis of type II toxin/antitoxin system in *Pseudomonas aeruginosa* isolates in exponential and stationary phases. J. Glob. Antimicrob. Resist..

[35] Coşkun U.S.Ş., Dagcioglu Y. (2023). Evaluation of toxin-antitoxin genes, antibiotic resistance, and virulence genes in *Pseudomonas aeruginosa* isolates. Rev. Assoc. Med. Bras. (1992).

[36] Lopes A.P.Y., Azevedo B.O.P., Emídio R.C., Damiano D.K., Nascimento A.L.T.O., Barazzone G.C. (2019). Analysis of Genetic VapC Profiles from the Toxin-Antitoxin Type II VapBC Modules among Pathogenic, Intermediate, and Non-Pathogenic. Microorganisms.

[37] Ren S.X., Fu G., Jiang X.G., Zeng R., Miao Y.G., Xu H., Zhang Y.X., Xiong H., Lu G., Lu L.F. (2003). Unique physiological and pathogenic features of *Leptospira interrogans* revealed by whole-genome sequencing. Nature.

[38] Trindade C.N.R., Panzenhagen P.H.N., Junqueira R.M., Silva D.C.V., Conte-Junior C.A., Balassiano I.T. (2020). Draft Genome Sequences of *Leptospira interrogans* Serovar Copenhageni Strains Isolated from Patients with Weil’s Disease in Brazil. Microbiol. Resour. Announc..

[39] Costa F., Hagan J.E., Calcagno J., Kane M., Torgerson P., Martinez-Silveira M.S., Stein C., Abela-Ridder B., Ko A.I. (2015). Global Morbidity and Mortality of Leptospirosis: A Systematic Review. PLoS Negl. Trop. Dis..

[40] Durski K.N., Jancloes M., Chowdhary T., Bertherat E. (2014). A global, multi-disciplinary, multi-sectorial initiative to combat leptospirosis: Global Leptospirosis Environmental Action Network (GLEAN). Int. J. Environ. Res. Public Health.

[41] Haake D.A., Levett P.N. (2015). Leptospirosis in humans. Curr. Top. Microbiol. Immunol..

[42] Lehmann J.S., Matthias M.A., Vinetz J.M., Fouts D.E. (2014). Leptospiral pathogenomics. Pathogens.

[43] Arcus V.L., McKenzie J.L., Robson J., Cook G.M. (2011). The PIN-domain ribonucleases and the prokaryotic VapBC toxin-antitoxin array. Protein Eng. Des. Sel..

[44] Senissar M., Manav M.C., Brodersen D.E. (2017). Structural conservation of the PIN domain active site across all domains of life. Protein Sci..

[45] Chauhan U., Barth V.C., Woychik N.A. (2022). tRNA. Antimicrob. Agents Chemother..

[46] Takahashi M.B., Teixeira A.F., Nascimento A.L.T.O. (2022). Host Cell Binding Mediated by. Int. J. Mol. Sci..

[47] Nascimento A.L., Verjovski-Almeida S., Van Sluys M.A., Monteiro-Vitorello C.B., Camargo L.E., Digiampietri L.A., Harstkeerl R.A., Ho P.L., Marques M.V., Oliveira M.C. (2004). Genome features of *Leptospira interrogans* serovar Copenhageni. Braz. J. Med. Biol. Res..

[48] Sonnhammer E.L., Eddy S.R., Durbin R. (1997). Pfam: A comprehensive database of protein domain families based on seed alignments. Proteins.

[49] Dienemann C., Bøggild A., Winther K.S., Gerdes K., Brodersen D.E. (2011). Crystal structure of the VapBC toxin-antitoxin complex from *Shigella flexneri* reveals a hetero-octameric DNA-binding assembly. J. Mol. Biol..

[50] Oppenheim D.S., Yanofsky C. (1980). Translational coupling during expression of the tryptophan operon of *Escherichia coli*. Genetics.

[51] Cataudella I., Trusina A., Sneppen K., Gerdes K., Mitarai N. (2012). Conditional cooperativity in toxin-antitoxin regulation prevents random toxin activation and promotes fast translational recovery. Nucleic Acids Res..

[52] Cooper C.R., Daugherty A.J., Tachdjian S., Blum P.H., Kelly R.M. (2009). Role of vapBC toxin-antitoxin loci in the thermal stress response of *Sulfolobus solfataricus*. Biochem. Soc. Trans..

[53] Nascimento A.L., Ko A.I., Martins E.A., Monteiro-Vitorello C.B., Ho P.L., Haake D.A., Verjovski-Almeida S., Hartskeerl R.A., Marques M.V., Oliveira M.C. (2004). Comparative genomics of two Leptospira interrogans serovars reveals novel insights into physiology and pathogenesis. J. Bacteriol..

[54] Lopes A.P., Lopes L.M., Fraga T.R., Chura-Chambi R.M., Sanson A.L., Cheng E., Nakajima E., Morganti L., Martins E.A. (2014). VapC from the leptospiral VapBC toxin-antitoxin module displays ribonuclease activity on the initiator tRNA. PLoS ONE.

[55] Ricaldi J.N., Fouts D.E., Selengut J.D., Harkins D.M., Patra K.P., Moreno A., Lehmann J.S., Purushe J., Sanka R., Torres M. (2012). Whole genome analysis of *Leptospira licerasiae* provides insight into leptospiral evolution and pathogenicity. PLoS Negl. Trop. Dis..

[56] Ramisetty B.C., Santhosh R.S. (2016). Horizontal gene transfer of chromosomal Type II toxin-antitoxin systems of *Escherichia coli*. FEMS Microbiol. Lett..

[57] Engelberg-Kulka H., Glaser G. (1999). Addiction modules and programmed cell death and antideath in bacterial cultures. Annu. Rev. Microbiol..

[58] Tchamedeu Kameni A.P., Couture-Tosi E., Saint-Girons I., Picardeau M. (2002). Inactivation of the spirochete recA gene results in a mutant with low viability and irregular nucleoid morphology. J. Bacteriol..

[59] Bhowmick A., Recalde A., Bhattacharyya C., Banerjee A., Das J., Rodriguez-Cruz U.E., Albers S.V., Ghosh A. (2024). Role of VapBC4 toxin-antitoxin system of. mBio.

[60] Khoo S.K., Loll B., Chan W.T., Shoeman R.L., Ngoo L., Yeo C.C., Meinhart A. (2007). Molecular and structural characterization of the PezAT chromosomal toxin-antitoxin system of the human pathogen *Streptococcus pneumoniae*. J. Biol. Chem..

[61] Ning D., Jiang Y., Liu Z., Xu Q. (2013). Characterization of a chromosomal type II toxin-antitoxin system mazEaFa in the Cyanobacterium Anabaena sp. PCC 7120. PLoS ONE.

[62] Ning D., Liu S., Xu W., Zhuang Q., Wen C., Tang X. (2013). Transcriptional and proteolytic regulation of the toxin-antitoxin locus vapBC10 (ssr2962/slr1767) on the chromosome of *Synechocystis* sp. PCC 6803. PLoS ONE.

[63] Yao J., Guo Y., Zeng Z., Liu X., Shi F., Wang X. (2015). Identification and characterization of a HEPN-MNT family type II toxin-antitoxin in *Shewanella oneidensis*. Microb. Biotechnol..

[64] Zheng C., Zhao X., Zeng T., Cao M., Xu J., Shi G., Li J., Chen H., Bei W. (2017). Identification of four type II toxin-antitoxin systems in Actinobacillus pleuropneumoniae. FEMS Microbiol. Lett..

[65] Harms A., Brodersen D.E., Mitarai N., Gerdes K. (2018). Toxins, Targets, and Triggers: An Overview of Toxin-Antitoxin Biology. Mol. Cell.

[66] Winther K.S., Gerdes K. (2011). Enteric virulence associated protein VapC inhibits translation by cleavage of initiator tRNA. Proc. Natl. Acad. Sci. USA.

[67] Starosta A.L., Lassak J., Jung K., Wilson D.N. (2014). The bacterial translation stress response. FEMS Microbiol. Rev..

[68] Christensen S.K., Mikkelsen M., Pedersen K., Gerdes K. (2001). RelE, a global inhibitor of translation, is activated during nutritional stress. Proc. Natl. Acad. Sci. USA.

[69] Silva J.C.A., Marques-Neto L.M., Carvalho E., Del Carpio A.M.G., Henrique C., Leite L.C.C., Mitsunari T., Elias W.P., Munhoz D.D., Piazza R.M.F. (2024). Chromosomal Type II Toxin-Antitoxin Systems May Enhance Bacterial Fitness of a Hybrid Pathogenic. Toxins.

